# Psychometric properties of the Brazilian Portuguese version of the Copenhagen Burnout Inventory (CBI) in healthcare professionals

**DOI:** 10.47626/2237-6089-2021-0362

**Published:** 2023-06-27

**Authors:** Carolina M. Moser, Bárbara Tietbohl-Santos, Daniel Luccas Arenas, Aurora Xavier, Felipe Ornell, Rogerio Boff Borges, Glen O. Gabbard, Pricilla B. Laskoski, Simone Hauck

**Affiliations:** 1 Programa de Pós-Graduação em Psiquiatria e Ciências do Comportamento Universidade Federal do Rio Grande do Sul Porto Alegre RS Brazil Programa de Pós-Graduação em Psiquiatria e Ciências do Comportamento, Universidade Federal do Rio Grande do Sul (UFRGS), Porto Alegre, RS, Brazil.; 2 Laboratório de Psiquiatria Psicodinâmica Hospital de Clínicas de Porto Alegre Porto Alegre RS Brazil Laboratório de Psiquiatria Psicodinâmica, Hospital de Clínicas de Porto Alegre (HCPA), Porto Alegre, RS, Brazil.; 3 Divisão de Promoção da Saúde Departamento de Atenção à Saúde UFRGS Porto Alegre RS Brazil Divisão de Promoção da Saúde, Departamento de Atenção à Saúde, UFRGS, Porto Alegre, RS, Brazil.; 4 Centro de Pesquisa em Álcool e Drogas HCPA UFRGS Porto Alegre RS Brazil Centro de Pesquisa em Álcool e Drogas, HCPA, UFRGS, Porto Alegre, RS, Brazil.; 5 Grupo de Pesquisa e Pós-Graduação Unidade de Bioestatística HCPA Porto Alegre RS Brazil Grupo de Pesquisa e Pós-Graduação, Unidade de Bioestatística, HCPA, Porto Alegre, RS, Brazil.; 6 Baylor College of Medicine Houston TX USA Baylor College of Medicine, Houston, TX, USA.

**Keywords:** The Copenhagen Burnout Inventory (CBI, reliability, validity, healthcare professionals, Brazil

## Abstract

**Introduction:**

Burnout syndrome (BS) in healthcare professionals (HCP) has been a major concern, and even more so during the coronavirus disease 2019 (COVID-19) pandemic. The need for adequate tools to assess BS is urgent. The objective of this study was to validate the Brazilian Portuguese version of the Copenhagen Burnout Inventory (CBI) in HCP.

**Methods:**

The sample comprised 1,054 Brazilian HCP. Data were collected for 1 month (May-2020 to June-2020) using an online self-administered questionnaire.

**Results:**

All three CBI dimensions demonstrated optimal reliability. All consistency measures attained values > 0.90. Split-half correlation values with Spearman-Brown reliability were higher than 0.8. The parallel analysis suggested two factors: personal burnout (PB) and work-related burnout (WB) items were associated with factor 1, and client-related burnout (CB) items were associated with factor 2.

**Conclusion:**

Our study corroborates the validity of the Brazilian Portuguese version of the CBI, pointing to a close relation between PB and WB in HCP. A public domain tool with evidence quality to ensure sufficient content validity can aid in burnout evaluation and encourage both expansion of the research field and accurate detection and treatment of this syndrome in Brazilian HCP.

## Introduction

Burnout syndrome (BS) has become a major concern among healthcare workers and students.^1 , 2^ Besides being considered an epidemic phenomenon in this population,^3 , 4^ BS has also been associated with a higher frequency of medical errors, reduced quality of life and empathy, suboptimal care of patients, absenteeism, and higher costs for health care systems.^2 , 5^ However, the lack of consensus regarding definitions and measures of burnout leads to a situation in which it is impossible to estimate its prevalence and, in fact, undermines the validity of the literature on the subject so far.^4^ Among the many problems is the fact that most research on burnout has been undertaken using the Maslach Burnout Inventory (MBI) and therefore, to an extent, “burnout is what the MBI measures, and the MBI measures what burnout is.”^6 , 7^

Nevertheless, many authors point out that there are many problems with the structure of the MBI. One of these is the fact that the “depersonalization” and “personal accomplishment” domains could be more accurately seen as a possible consequence of the syndrome than as part of the core burnout concept.^4^ A recent study aiming at harmonizing the definition of occupational burnout through a systematic review followed by semantic analysis and expert consensus building has proposed that “in a worker, occupational burnout or occupational physical AND emotional exhaustion state is an exhaustion due to prolonged exposure to work-related problems.”^6^ Therefore, physical AND emotional exhaustion can be considered the core of BS.

Additionally, Shoman et al.^8^ have recently published a systematic review that evaluated the psychometric validity of five of the most common Patient-Reported Outcome Measures (PROMs) that assess burnout symptoms, including the MBI. Among the evaluated PROMs, the Copenhagen Burnout Inventory (CBI) along with the Oldenburg Burnout Inventory (OLBI) emerged as the only ones with sufficient quality evidence of content validity,^8^ thus corroborating the need for burnout assessment with more valid instruments such as the CBI.

Kristensen et al. developed the CBI in 2005^7^ to be a more straightforward measure that considers fatigue and exhaustion as the core constructs of burnout, aiming to resolve some of the problems observed in previous questionnaires such as the MBI. Besides, the CBI is a public domain tool that evaluates the same overall construct in different contexts. The CBI has been translated, validated, and used in many countries with a growing evidence base of good psychometric properties.^7 , 9 - 12^ A Brazilian Portuguese version has already been validated in students.^13^ However, to the best of our knowledge, there are no studies evaluating the CBI’s psychometric properties in healthcare professionals (HCP). In the midst of the COVID-19 pandemic, early and accurate detection of burnout signs becomes even more important. The aim of this study was to investigate the reliability and validity of the Brazilian Portuguese version of the CBI in Brazilian HCP.

## Methods

### Participants

The total sample comprised 1,054 HCP working in Brazil: 34.5% were physicians (n = 364), 19.1% were nursing technicians (n = 201), 14.2% were nurses (n = 150), 12% were psychologists (n = 126), and 19.3% were other healthcare workers (n = 213). Subjects with incomplete or missing questionnaires were excluded.

### Measure

Burnout level was assessed with the Brazilian Portuguese version of the CBI adapted for HCP (Supplementary Material S1). The Brazilian CBI version for students, as previously validated by Campos et al.,^13^ was adjusted for HCP through a standardized protocol developed by our research group and described elsewhere.^14^ The protocol was developed in compliance with both the International Society for Pharmacoeconomics and Outcomes Research (ISPOR) Task Force’s Principles of Good Practice for the Translation and Cultural Adaptation Process for Patient-Reported Outcomes^15^ and the European Regulatory Issues on Quality of Life Assessment Group (ERIQ-A)’s advice towards a multistep approach.^16^

The CBI has three sub-dimensions: personal burnout (PB) (the degree to which a person perceives her or himself as physically and psychologically exhausted), work-related burnout (WB) (the degree to which physical and psychological exhaustion is perceived concerning work activities), and client-related burnout (CB) (the level of exhaustion that a person perceives that stems from the professional relationship with clients). The WB questions assume that the respondent has paid work of some kind and the client-related dimension implies that the respondent works with people.

The CBI is a self-report 19-item questionnaire that measures three sub-dimensions that can be used independently: PB (six items), WB (seven items), and CB (six items). It uses a five-point Likert response scale: “Always” or “To a very high degree” (100 points), “Often” or “To a high degree” (75 points), “Sometimes” or “Somewhat” (50 points), “Seldom” or “To a low degree” (25 points), and “Never/almost never” or “To a very low degree” (0 points). The CBI items in each subscale are summed and averaged to obtain the scores. The higher the score, the higher the level of burnout.^7^

### Procedure

This is an instrument validation study. Participants were recruited with an online snowball method via email and social media targeting Brazilian HCP for 1 month (May 2020 to June 2020). The questionnaire was made available on a platform widely used for research purposes because it guarantees the anonymity of the subjects involved (SurveyMonkey™).

### Ethical consideration

Data collection was initiated after approval was granted by the Hospital de Clínicas de Porto Alegre Ethics Committee (CAAE 30745020.5.0000.5327). Acceptance and completion of the questionnaire occurred entirely online and all participants agreed to participation, providing online informed consent.

### Data analyses

The reliability of each dimension was assessed using ordinal coefficient alpha and split-half correlation with Spearman-Brown reliability.

Even though the purpose of the instrument is to evaluate the presence of burnout in each dimension independently, we decided to perform a factor analysis to explore how the items from the distinct dimensions would relate with each other in our sample of Brazilian HCP. The decision to perform an exploratory analysis was taken because the CBI has never been tested in this population before. Furthermore, the recent debate regarding the BS construct in the literature points clearly to the importance of deepening understanding of the burnout phenomena in different populations and cultures.

The suitability of data for factorization was assessed with the Kaiser-Meyer-Olkin (KMO) measure and Bartlett’s test of sphericity. The exploratory factor analysis employed oblique rotation. The number of factors was determined using the parallel analysis method. All of these analyses were performed using the polychoric correlation between items. The analyses were performed in the R program using the psych version 2.1.9 and multicon version 1.6 packages.^17 - 19^ Where necessary, a 5% significance level was adopted.

## Results

The CBI presented very good reliability. All consistency measures showed values > 0.90. The split-half correlation values with Spearman-Brown reliability were higher than 0.8. Table 1 shows both the results for each of the three dimensions independently and the results for the two factors that emerged from the factor analysis.

**Table 1:** The split-half correlation values with Spearman-Brown reliability

	Cronbach		Split-half correlation		
	
Dimension	Alpha	95%CI	Correlation	Reliability	SD
PB	0.9215	0.9137-0.9285	0.8391	0.9125	0.0801
WB	0.8910	0.8804-0.9006	0.7795	0.8761	0.0792
CB	0.9296	0.9227-0.9359	0.8382	0.912	0.0867
PB + WB	0.9457	0.9407-0.9503	0.8837	0.9382	0.0757
CB	0.9296	0.9227-0.9359	0.8382	0.912	0.0867

95%CI = 95% confidence interval; SD = standard deviation.

Copenhagen Burnout Inventory (CBI) sub-dimensions: CB = client-related burnout; PB = personal burnout; WB = work-related burnout.

The KMO measure was 0.95 and Bartlett’s test of sphericity rejected the null hypothesis (p < 0.001). The parallel analysis suggested two factors (Supplementary Material S2): the items comprising the PB and WB dimensions of the CBI were associated with factor 1 and the items comprising the CB dimension were associated with factor 2.

## Discussion

The present study aimed to investigate the reliability and validity of the Brazilian Portuguese version of the CBI in Brazilian HCP. Our results suggest that the Brazilian Portuguese version of the CBI is a reliable and valid instrument for measuring burnout in HCP. All CBI domains had excellent internal consistency reliability indexes, slightly higher than those obtained by Kristensen et al.^7^ and similar to those found for the previously validated Brazilian Portuguese CBI version for students.^13^

The fact that our factor analysis suggested two factors is aligned with other CBI studies with HCP that have shown good discriminant validity of the subscales, except between the PB and WB.^11 , 12 , 20 , 21^ One possible explanation is an essential overlap between PB and WB in HCP. Our findings also corroborate use of the PB dimension alone as suggested by the authors of the instrument^7^ in contexts where it is more suitable, such as epidemiological studies or for screening.

Our study’s strength is its recognition of the complexity of different cultures, providing a careful cross-cultural adaptation for the intended population along with a validation study. Furthermore, our findings were based on a PROM that has sufficient quality evidence of content validity^8^ to assess burnout symptoms. Nevertheless, our study has some limitations, since it was based on an online convenience sample. Therefore, the external validity of these findings should be confirmed with different samples of HCP. Also, other methods of analysis could be applied in the future, including test-retest reliability.

Assessment of burnout symptoms using valid instruments is a priority. As far as we know, this is the first study to evaluate the validity of the Brazilian Portuguese version of the CBI in a sample of HCP. The consistency of our findings provides a valid, reliable, and accessible public domain tool that can foster research on burnout in Brazilian HCP and provide a screening and follow-up instrument for clinical purposes.
