# Supplementary material for: Psychometric properties of the Brazilian Portuguese version of the Copenhagen Burnout Inventory (CBI) in healthcare professionals
Source: Trends Psychiatry Psychother. 2023 Jun 27;45:e20210362. doi: 10.47626/2237-6089-2021-0362 (PMC10416248; doi:10.47626/2237-6089-2021-0362)
Supplement: Supplementary file 1 [file 2238-0019-trends-45-e20210362-suppl1.pdf]

http://doi.org/10.47626/2237-6089-2021-0362

Supplementary Material S1 - Brazilian Portuguese version of The Copenhagen Burnout Inventory (CBI) adapted for healthcare professionals

Burnout Pessoal

PB1 **Com que frequência você se sente cansado (a)?**
❑ Sempre
❑ Frequentemente
❑ Às vezes
❑ Raramente
❑ Nunca

PB2 **Com que frequência você fica exausto (a) fisicamente?**
❑ Sempre
❑ Frequentemente
❑ Às vezes
❑ Raramente
❑ Nunca

PB3 **Com que frequência você fica exausto (a) emocionalmente?**
❑ Sempre
❑ Frequentemente
❑ Às vezes
❑ Raramente
❑ Nunca

PB4 **Com que frequência você pensa: "Eu não aguento mais"?**
❑ Sempre
❑ Frequentemente
❑ Às vezes
❑ Raramente
❑ Nunca

PB5 **Com que frequência você se sente esgotado (a)?**
❑ Sempre
❑ Frequentemente
❑ Às vezes
❑ Raramente
❑ Nunca

PB6 **Com que frequência você se sente fraco (a) e suscetível à doença?**
❑ Sempre
❑ Frequentemente
❑ Às vezes
❑ Raramente
❑ Nunca

Burnout relacionado ao trabalho

WB1 **Você se sente esgotado (a) no fim de um dia de trabalho?**
❑ Sempre
❑ Frequentemente
❑ Às vezes
❑ Raramente
❑ Nunca

WB2 **Você fica exausto (a) pela manhã ao pensar em mais um dia de trabalho?**
❑ Sempre
❑ Frequentemente
❑ Às vezes
❑ Raramente
❑ Nunca

WB3 **Você se sente mais cansado a cada hora de trabalho?**
❑ Sempre
❑ Frequentemente
❑ Às vezes
❑ Raramente
❑ Nunca

WB4 **Você tem energia suficiente para família e amigos durante os momentos de lazer?**
❑ Sempre
❑ Frequentemente
❑ Às vezes
❑ Raramente
❑ Nunca

WB5 **O seu trabalho é exaustivo emocionalmente?**
❑ Em um grau muito alto
❑ Em um grau alto
❑ Em algum grau
❑ Em baixo grau
❑ Em um grau muito baixo

WB6 **O seu trabalho lhe frustra?**
❑ Em um grau muito alto
❑ Em um grau alto
❑ Em algum grau
❑ Em baixo grau
❑ Em um grau muito baixo

WB7 **Você se sente esgotado por causa do seu trabalho?**
❑ Em um grau muito alto
❑ Em um grau alto
❑ Em algum grau
❑ Em baixo grau
❑ Em um grau muito baixo

<u>Burnout relacionado aos pacientes</u>
CB1 **Você acha difícil trabalhar com pacientes?**
❑ Em um grau muito alto
❑ Em um grau alto
❑ Em algum grau
❑ Em baixo grau
❑ Em um grau muito baixo

CB2 **Trabalhar com pacientes suga a sua energia?**
❑ Em um grau muito alto
❑ Em um grau alto

❏ Em algum grau
❏ Em baixo grau
❏ Em um grau muito baixo

CB3 **Você acha frustrante trabalhar com pacientes?**
❏ Em um grau muito alto
❏ Em um grau alto
❏ Em algum grau
❏ Em baixo grau
❏ Em um grau muito baixo

CB4 **Você sente que está dando mais do que recebe quando você trabalha com pacientes?**
❏ Em um grau muito alto
❏ Em um grau alto
❏ Em algum grau
❏ Em baixo grau
❏ Em um grau muito baixo

CB5 **Você está cansado (a) de trabalhar com pacientes?**
❏ Sempre
❏ Frequentemente
❏ Às vezes
❏ Raramente
❏ Nunca

CB6 **Você às vezes se pergunta quanto tempo será capaz de continuar trabalhando com pacientes?**
❏ Sempre
❏ Frequentemente
❏ Às vezes
❏ Raramente
❏ Nunca

Supplementary Material S2 - Factor analysis of the Copenhagen Burnout Inventory (CBI)

Item	Factor 1	Factor 2
PB1	0.800	-0.051
PB2	0.860	-0.094
PB3	0.809	-0.003
PB4	0.834	0.014
PB5	0.910	-0.032
PB6	0.711	0.005
WB1	0.859	-0.054
WB2	0.722	0.120
WB3	0.743	0.049
WB4	-0.558	-0.103
WB5	0.479	0.166
WB6	0.365	0.385
WB7	0.678	0.236
CB1	-0.075	0.883
CB2	0.013	0.860
CB3	-0.060	0.930
CB4	0.131	0.659
CB5	0.056	0.834
CB6	0.175	0.661

Data presented as polychoric correlations between the items.
CBI sub-dimensions: CB(x) = client-related burnout; PB(x) = personal burnout; WB(x) = work-related burnout.
Factor 1 = personal and work-related fatigue and exhaustion; Factor 2 = exhaustion which is perceived by the person as related to client/patient work.
